# Computed tomography-based volumetric tool for standardized measurement of the maxillary sinus

**DOI:** 10.1371/journal.pone.0190770

**Published:** 2018-01-05

**Authors:** Guilherme Giacomini, Ana Luiza Menegatti Pavan, João Mauricio Carrasco Altemani, Sergio Barbosa Duarte, Carlos Magno Castelo Branco Fortaleza, José Ricardo de Arruda Miranda, Diana Rodrigues de Pina

**Affiliations:** 1 Instituto de Biociências de Botucatu, Universidade Estadual Paulista (IBB-UNESP), Botucatu, São Paulo, Brazil; 2 Hospital de Clínicas, Universidade Estadual de Campinas (HC-UNICAMP), Campinas, São Paulo, Brazil; 3 Centro Brasileiro de Pesquisas Físicas (CBPF), Rio de Janeiro, Rio de Janeiro, Brazil; 4 Faculdade de Medicina de Botucatu, Universidade Estadual Paulista (FMB-UNESP), Botucatu, São Paulo, Brazil; Kaplan Medical Center, ISRAEL

## Abstract

Volume measurements of maxillary sinus may be useful to identify diseases affecting paranasal sinuses. However, literature shows a lack of consensus in studies measuring the volume. This may be attributable to different computed tomography data acquisition techniques, segmentation methods, focuses of investigation, among other reasons. Furthermore, methods for volumetrically quantifying the maxillary sinus are commonly manual or semiautomated, which require substantial user expertise and are time-consuming. The purpose of the present study was to develop an automated tool for quantifying the total and air-free volume of the maxillary sinus based on computed tomography images. The quantification tool seeks to standardize maxillary sinus volume measurements, thus allowing better comparisons and determinations of factors that influence maxillary sinus size. The automated tool utilized image processing techniques (watershed, threshold, and morphological operators). The maxillary sinus volume was quantified in 30 patients. To evaluate the accuracy of the automated tool, the results were compared with manual segmentation that was performed by an experienced radiologist using a standard procedure. The mean percent differences between the automated and manual methods were 7.19% ± 5.83% and 6.93% ± 4.29% for total and air-free maxillary sinus volume, respectively. Linear regression and Bland-Altman statistics showed good agreement and low dispersion between both methods. The present automated tool for maxillary sinus volume assessment was rapid, reliable, robust, accurate, and reproducible and may be applied in clinical practice. The tool may be used to standardize measurements of maxillary volume. Such standardization is extremely important for allowing comparisons between studies, providing a better understanding of the role of the maxillary sinus, and determining the factors that influence maxillary sinus size under normal and pathological conditions.

## Introduction

The paranasal sinuses are complex anatomical structures with significant interindividual variations [1, 2].The actual function of the maxillary sinus (MS) is largely unknown [3]. Previous studies have suggested that the MS lessens the weight of the skull, confers resonance to speech, and warms and moistens inspired air [3, 4]. In this context, volumetric measurements provide important information about the MSs [5, 6]. Comprehensive knowledge of MS development is important for elucidating sinus pathologies and selecting adequate treatments [7]. Computed tomography (CT) is the most comprehensive and objective method of measuring disease severity and is very sensitive to changes that are caused by different interventions [7–9].

In clinical practice, evaluation based on air-free or modified mucosa volume of the sinuses may help the diagnosis and management of sinusopathies [2, 10–14]. For example, volumetric evaluation contributes to the assessment of patients with chronic rhinosinusitis (CRS) because such a volumetric method better correlates with the severity of CRS compared with most widely used Lund-Mackay CT scoring system [10]. Furthermore, MS volume have been evaluated for verifying the response to chemo- and radiotherapy in malignant tumors [11] and for planning procedures that involve sinus floor elevation in the placement of implants [12, 13] and endoscopic sinus surgery [2, 14].

The size and shape of the MS has been investigated in many previous studies [1]. The literature shows the influence of breathing patterns [4, 15], dental problems [7, 16], anatomical features [2, 16, 17], gender [1, 2, 18], age [18, 19], ethnicity [20, 21], and climatic factors [22, 23] on MS volume. Furthermore, many chronological and pathological events can affect MS volume.

Studies in literature varied in relation to measurements devices and study objectives [24], thereby causing a lack of consensus regarding MS volume results [11, 25]. Among other reasons, this may be attributable to different computed tomography data acquisition techniques, segmentation methods, and focuses of investigation [25]. Manual and semiautomated methods are commonly used for the quantification of MS volume for pathological sinuses [5, 23, 26, 27], however, such task is labor intensive. Automated techniques may reduce both time and effort and provide relatively quick and easy segmentation, more accuracy, better control, and higher sensitivity [4].

The purpose of the present study was to develop an automated tool to quantify the total and air-free volume of the MS using CT images. The tool can quantify sinus volume even under pathological conditions, such as tumors and rhinosinusitis, thus contributing to the clinical management of patients. Therefore, the methodology that was developed herein may potentially replace the current radiologic scores, such as the Lund-Mackay CT scoring system, because of its accuracy in MS volumetry. Furthermore, the present automated tool seeks to standardize MS volume measurements.

## Methodology

### Patient selection

This retrospective study was developed with ethical approval from the authors’ institutions and national review panels (protocol no. CAAE 42225115.4.0000.5411). The study involved 30 patients who were randomly selected from a pool of 287 patients who were treated in the Hospital das Clínicas de Botucatu, Brazil, between January 2013 and December 2015. Paranasal sinus CT exams that were evaluated in the present study were indicated for patients with clinically suspected rhinosinusitis and septal deviation. The gender distribution was predominantly male (16 [53.3%]). The mean age of the patients was 28.4 ± 5.2 years.

The inclusion criteria were patients older than 20 years who underwent a paranasal sinus CT exam. Patients with a history of previous nasal, nasopharyngeal, paranasal sinus, or adenoidectomy surgery, maxillofacial trauma, and congenital nasal abnormalities were excluded from the study.

### Data acquisition

Paranasal sinus CT exams without contrast enhancement were evaluated in the study. All the CT scans were acquired using a Toshiba Activion 16 Helicoidal device (Toshiba America Medical Systems, Tustin, CA, USA) with the following parameters: pixel size range of 0.30 mm × 0.30 mm to 0.38 mm × 0.38 mm, 512 × 512 pixel matrix, 3.0 mm increment between slices, 3.0 mm slice width, and 120 kV tube voltage. Volumetric reconstructions were made from raw data using a 0.5 mm slice width and 0.3 mm interval between slices.

### Automated tool

An automated algorithm was developed using Matlab R2013a (Mathworks, Natick, MA, USA) for the volumetric quantification of the MS based on CT exams. The algorithm is available in the protocols.io repository (dx.doi.org/10.17504/protocols.io.ig7cbzn) and in website GitHub (https://github.com/dianapina/Maxillary-Sinus-Quantification.git).

**Fig 1:**
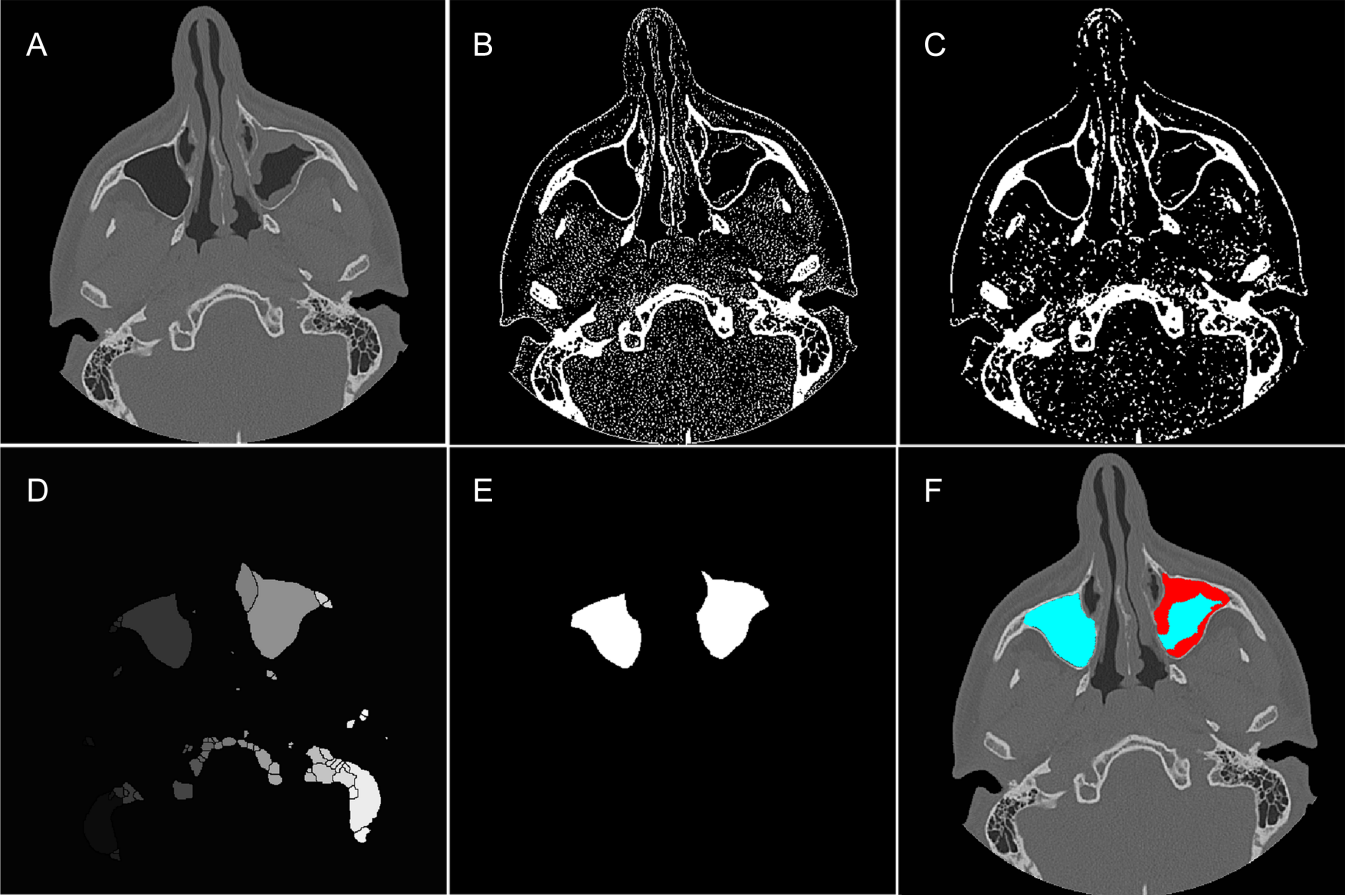
Tool for automated maxillary sinus segmentation. (A) Original image. (B) Image after thresholding process. (C) Thresholded image after the application of morphological operators. (D) Partitioned image after watershed technique, where each gray level represents different segmented regions. (E) Final segmentation of the MS after applying the rule-based system. (F) Segmentation of the MS, highlighting air-free (blue) and involvement (red) areas.

It was initially necessary to detect and segment MS areas in the CT exams. Fig 1 shows this process in one CT slice using a hybrid methodology. The algorithm is defined as the following:

1. CT image was read (Fig 1A).
2. The original image was thresholded to remove soft tissue and mucous membrane thickening, cysts, and/or fluid, highlighting bone regions from the MS (Fig 1B). In this step, the threshold was 150 Hounsfield Units (HU).
3. The resulting thresholded image was then fine-tuned using morphological image processing operators [28]. In this step, an opening operation (i.e., erosion followed by dilation) was applied to fill the boundaries of the MS and remove small-sized areas (Fig 1C) [29], thus automatically reducing sparse voxels that were attributable to imaging noise [28].
4. The watershed technique was applied, which can be classified as a region-based segmentation approach [30, 31]. This step computes a complete partition of the image into basins. The watershed was then determined by boundary detection. Fig 1D shows segmentation by the watershed technique, in which each gray level represents different basins.
5. A rule-based system was applied to compare the major areas by assessing data on the position, shape, and symmetry of the segmented regions (basins) and selecting only the MS areas (Fig 1E).
6. The areas of free-air and involvement (mucous membrane thickening, cysts, and/or fluid) were classified (Fig 1F; represented by blue and red regions, respectively). This process was performed using the threshold technique. The range of attenuation of air in the MS was set between -200 and -1200 HU [1].

**Fig 2:**
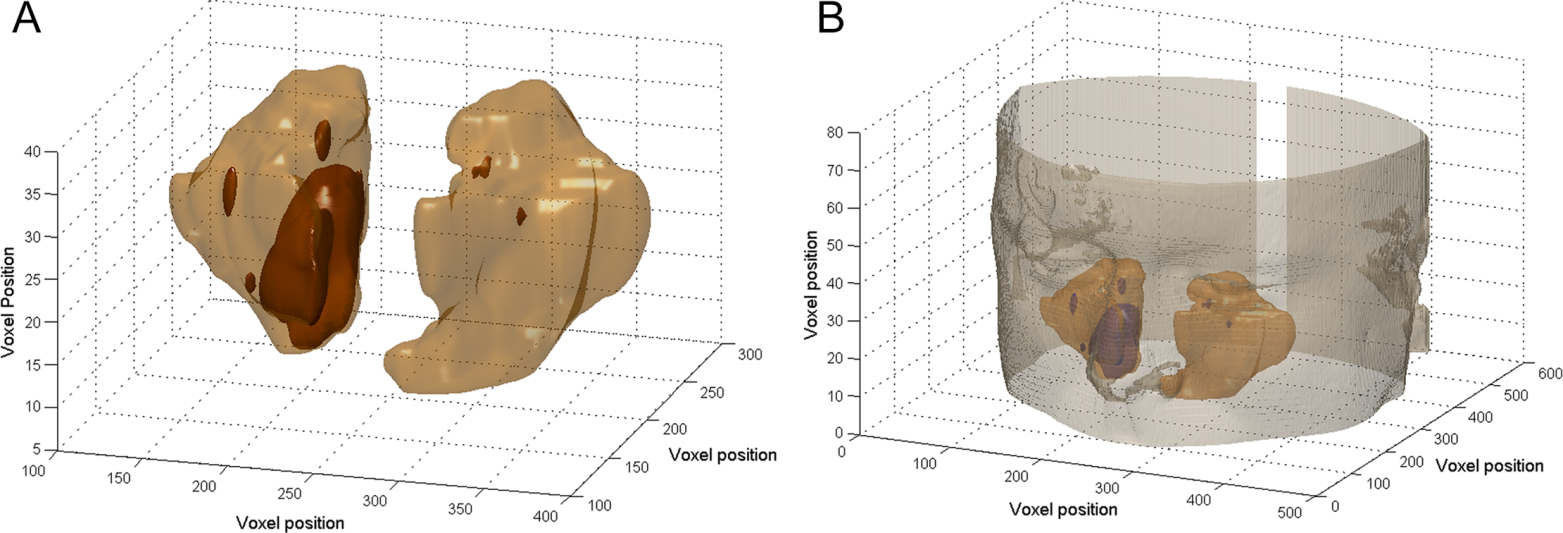
Reconstruction of three-dimensional shaded surface of the maxillary sinuses (beige surface), highlighting maxillary sinus involvement (brown surface). (A) Maxillary sinuses. (B) Maxillary sinuses inside reconstructed head.

The methodology described above was initially applied to the middle slice of the CT exam. The immediately lower and upper slices were also evaluated using this methodology. This process was repeated until the rule-based system was no longer satisfied. Thus, the entire CT exam was assessed, resulting in a volumetric region of interest. The total and air-free volumes of the MS were measured by multiplying the number of voxels in the volumetric region by the voxel volume, which could be reconstructed into a three-dimensional (3D) image [16]. The volume of both MSs was calculated in all of the CT exams. Fig 2A illustrates the 3D shaded surface of the MS, highlighting MS involvement. The MS inside the reconstructed head is shown in Fig 2B.

### Validation by manual segmentation

**Fig 3:**
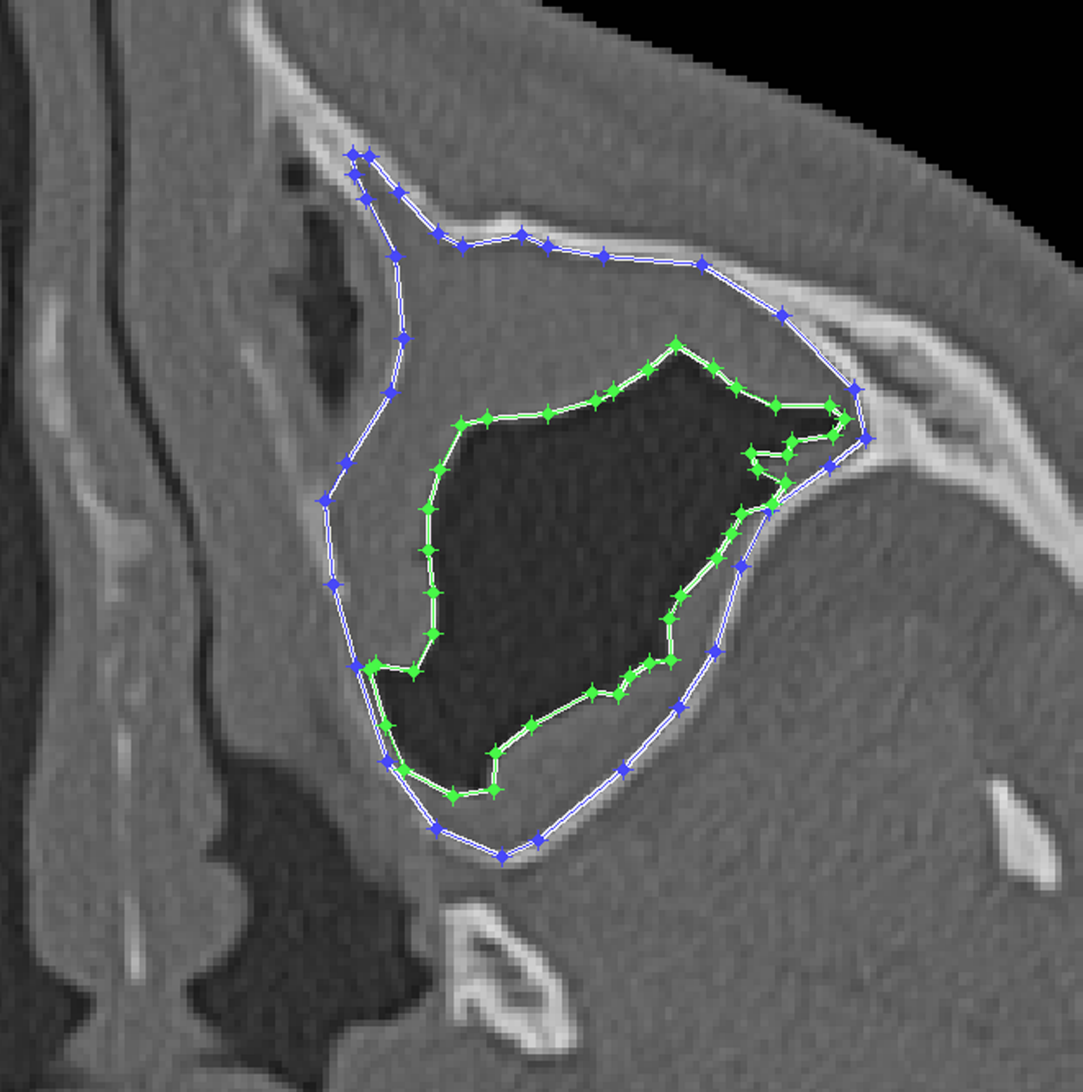
Example of the process of manual segmentation of the left maxillary sinus for one slice. Blue line, maxillary sinus. Green line, air-free areas.

To evaluate the accuracy of the automated MS volume quantification based on CT images, the results from the same 30 patients were compared with manual segmentation that was performed by an experienced radiologist (J.M.C.A.) using a standard procedure. Each MS was segmented by carefully tracing the outlines of the MS while following the inner bone surface, proceeding in an axial direction [11]. In MSs with involvement, the air-free area was also segmented. The MS areas were segmented in all slices of the CT exam, thus defining a volumetric region of interest. The total and air-free volumes of the MS were measured by multiplying the number of voxels in the volumetric region by the voxel volume, which was obtained from the DICOM header of the CT images. All of the data were measured in cm^3^, and the measurements were performed by the same radiologist to prevent possible interobserver variability [27]. An example of the left MS manual segmentation process of one slice is shown in Fig 3. In this step, the radiologist segmented the MS boundaries (blue line) and free-air areas (green line).

Statistical linear regressions and mean percent differences between the automated and manual methods were performed for the same CT exam. Comparisons between volume quantifications were performed using Bland-Altman statistics [32] to assess agreement between the presently developed algorithm and the reference standard, with a confidence interval of 95%.

## Results

The automated volume quantification method that was developed herein and manual segmentation method were compared based on the same 30 patient examinations, for a total of 60 MSs. An average total MS volume of 14.7 ± 4.4 cm^3^ was found in the evaluated patients. The raw data for the quantification are presented in S1 Table.

The mean percent difference between both methods was 7.19% ± 5.83% and 6.93% ± 4.29% for total and air-free MS volumes, respectively.

**Fig 4:**
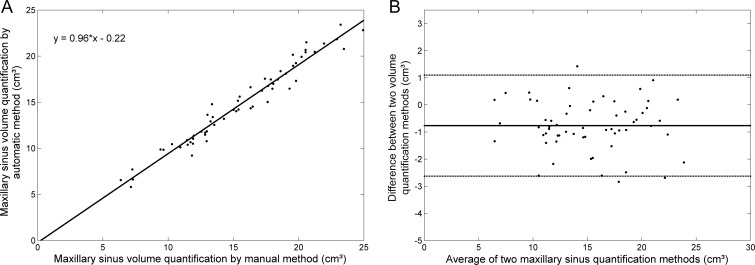
Total maxillary sinus volume quantification agreement between the automated segmentation method developed herein and the manual segmentation method (reference standard) for 60 maxillary sinuses from 30 computed tomography exams. (A) Linear regression. y = 0.96x -0.22, *R^2^* = 0.96. (B) Bland-Altman plot for both quantification methods. The difference refers to the automated method minus the reference standard. The central line corresponds to the mean value of deviation. The dashed lines indicate the interval of 2 standard deviations.

For total MS volume quantification, the linear regression (Fig 4A) was y = 0.96x – 0.22, with a correlation coefficient of *R^2^* = 0.96. The statistical Bland-Altman plot is shown in Fig 4B for the difference and average between the automated and manual methods.

**Fig 5:**
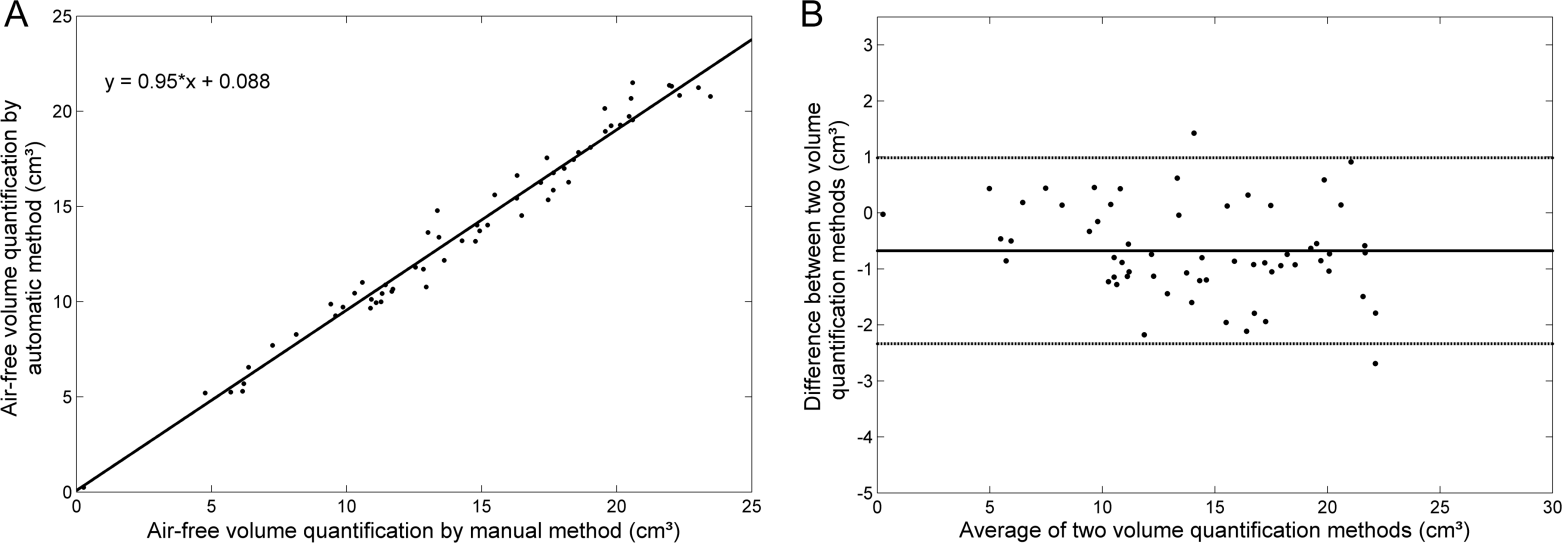
Air-free volume quantification agreement between the automated segmentation method developed herein and the manual segmentation method (reference standard) for 60 maxillary sinuses from 30 computed tomography exams. (A) Linear regression. y = 0.95x + 0.088, *R^2^* = 0.98. (B) Bland-Altman plot for both quantification methods. The difference refers to the automated method minus the reference standard. The central line corresponds to the mean value of deviation. The dashed lines indicate the interval of 2 standard deviations.

For air-free MS quantification, the linear regression (Fig 5A) was y = 0.95x + 0.088, with a correlation coefficient of *R^2^* = 0.98. The statistical Bland-Altman plot is shown in Fig 5B.

Figs 4 and 5 show good agreement between the automated and manual methods for both total MS volume and air-free volume within the MS. The Bland-Altman analyses showed no significant differences. For total MS volume (Fig 4B), the bias was -0.77 cm^3^, with -2.63 cm^3^ and 1.10 cm^3^ as limits of agreement at a 95% confidence interval. For MS air-free volume (Fig 5B), the bias was -0.68 cm^3^, with -2.34 cm^3^ and 0.99 cm^3^ as limits of agreement at a 95% confidence interval.

## Discussion

The size of the human paranasal sinus was initially determined by taking anatomical measurements, injecting different materials into cadavers, or performing plain radiography [11, 23, 33]. The introduction of CT and magnetic resonance imaging has allowed more precise assessments of these structures [1, 11, 15]. CT is widely used for evaluating the volume of the paranasal sinuses [1, 7, 9, 10, 34]. Although magnetic resonance imaging is superior to CT in rendering soft tissue, its use is limited by its relatively high cost and restricted accessibility [15]. The major advantage of CT is the excellent osseous anatomic detail that it provides, highlighting the boundaries of the MS. Therefore, CT is the gold-standard imaging modality for inflammatory diseases of the paranasal sinuses [9, 34].

Previous studies have evaluated gender differences in MS volume, in which MS volume is significantly larger in males than in females [1, 2, 18]. However, Pirner *et al*.reported no significant differences between male and female MS volumes [5]. Some studies have shown that MS volume is significantly less in patients with chronic rhinosinusitis compared with controls [16, 33]. However, Fernández *et al*. showed that MS volumes in chronic rhinosinusitis patients were larger than in the control group [11]. Other studies have evaluated the influence of dentition status on MS volume. Cho *et al*. found that a group of patients with abnormal teeth presented no difference in MS volume compared with patients with normal teeth [16]. In contrast, Möhlhenrich *et al*. found that dentition status influenced the volume of the MS [26].Because of the lack of a gold-standard technique for quantifying the volume of the paranasal sinuses, no consensus has been reached regarding such measurements [11, 24]. These studies also did not take into account possible influences of instrumental, physical, and human limitations that may exist, which could cause MS volume measurements to be different from the actual value [11, 25].

In clinical practice, CT-based sinus volumetry has been reported to be a useful tool for objectively evaluating sinus disease [2, 10–14]. Pallanch *et al*. evaluated the total percent volume of sinus disease based on CT and the Lund-Mackay scoring system in patients who were being medically treated for CRS. Volumetric scoring using CT exams had a better correlation with disease severity (i.e., symptoms, endoscopic scoring, and quality of life) compared with Lund-Mackay scoring. These results show that sinus volumetry can contribute to the clinical management of CRS patients. However, as indicated by these authors, a tool is still needed to automatically segment sinus volume to reduce the effort that is required for manual segmentation [10].

To our knowledge, only manual or semiautomated methods have been applied for paranasal sinus segmentation. These techniques present inter- and intraobserver variability and are both time-consuming. Deeb *et al*. attempted to perform 3D volumetric measurements based on CT scans of the MS in patients with chronic rhinosinusitis using image analysis software. However, their method was too cumbersome to evaluate the whole sinus in a large number of patients [28]. Kirmeier *et al*. tested a semiautomated volumetric analysis technique for MS quantification. They achieved good results with their time-consuming measurement procedure, supporting its applicability for clinical evaluations of small changes in MS volume following sinus augmentation or tooth extraction. However, they concluded that a reasonable goal would be to develop a fully automated sinus volume determination technique with high accuracy [25]. Thus, CT-based volume determinations can currently be performed accurately and effectively, but a fully automated method may have better applicability in clinical practice [7].

In the present study, we developed an automated method for MS volume measurements based on CT images. The statistical comparisons between the automated and manual quantification methods revealed strong agreement and low dispersion between variables. These promising findings were maintained over the entire range of MS volume evaluation, with no increase in quantification error as the MS volume varied between patients. Furthermore, the mean percent difference between the automated and manual techniques was approximately 7% for both measurements. These differences were sufficiently small to yield the same level of confidence for both quantification methods.

The average total MS volume found was within the range of published mean volumetric measurements for adult patients (10.9 ± 2.8 cm^3^ [4] to 24.7 ± 9.0 cm^3^ [28]). Importantly, our database was composed of relatively young patients (mean age, 28.4 ± 5.2 years). Some studies have reported an age-related reduction of volume [6, 18, 19]. Our volumetric findings for the MS within the same age range are similar to the results that were reported by Jun *et al*. (18.6 ± 7.8 cm^3^) [19] and Park *et al*. (14.8 ± 1.5 cm^3^) [35]. However, Kawarai *et al*. evaluated patients with a mean age of 29.5 years and reported volumes of 21.3 ± 6.5 cm^3^ and 23.1 ± 6.7 cm^3^ for the right and left MSs, and these volumes were greater than those found in the present study [6, 19].

The combination of different image processing techniques using a fully automated hybrid method indicates the novelty of this study. Our method was shown to accurately quantify MS volume, including both total and air-free volumes. The tool allows comprehensive assessments of the MS while taking into account MS involvement, which is determined based on the relationship between air-free and total volume. In pathological sinuses, defining pixel intensity is difficult because of their non-homogeneous constitutions of bone, air, and mucosa. This occurs because of the anatomical complexity of the paranasal sinuses [5]. However, the tool was shown to be suitable for quantifying the volume of involvement in pathological sinuses and evaluating mucous membrane thickening, cysts, fluid, tumors, and other materials with different densities. This is a powerful advantage because the available methods for MS volume quantification are unsuitable for pathological sinuses [5].

Advances in multislice CT equipment have enabled high-resolution scans with consequently high structural definition, mainly due to the ability to produce exams with several thin slices [36]. Since the MS is imaged using ~120 slices per exam, manual segmentation requires approximately 2 hours to complete because of the required per-slice user interaction. The present automated tool was able to quantify MS volume in approximately 3 minutes per exam, showing to be less time-consuming. Furthermore, the presently developed tool does not require complex or expensive equipment. The algorithm may be applied using conventional computers, thus allowing better implementation in clinical practice.

The present study has some limitations. Our methodology was analyzed using only one protocol with a slice thickness of 0.5 mm. Prionas *et al*.[37] reported a greater error of volume quantification for thicker slices. Moreover, our objective was to validate our tool, and the MS volumes that are presented herein should not be considered a cohort study. Further studies are needed to evaluate MS volume in patient groups with different ages, genders, and ethnicities. The study is limited to the MS, and therefore its role in functional endoscopy surgery is yet to be proven. Nevertheless, the automated tool may be adapted to quantify volume in other paranasal sinuses.

In conclusion, the present study found a good correlation between the manual and automated MS volume estimation techniques. Our automated measurements of MS volume based on CT exams were reliable, robust, and accurate compared with the manual method. Our findings suggest that this automated tool may be applied in clinical practice. It does not require substantial user expertise, and it is reproducible and fast. Our tool may allow comparisons between different patient groups by standardizing measurements of MS volume while obviating the variability that is inherent in different measurement procedures. Such standardization is extremely important for comparisons between studies. The tool may also be applied to determine the factors that influence MS pneumatization. Furthermore, MS volumetry using our method is feasible even under pathological conditions, which might contribute to a better clinical assessment of the extent of nasal pathology.

## Supporting information

S1 Table Raw data for the maxillary sinus volumetry by automatic and manual quantifications. (DOCX)
