# Supplementary material for: Computed tomography-based volumetric tool for standardized measurement of the maxillary sinus
Source: PLoS One. 2018 Jan 5;13(1):e0190770. doi: 10.1371/journal.pone.0190770 (PMC5755892; doi:10.1371/journal.pone.0190770)
Supplement: S1 Table — (DOCX) [file pone.0190770.s001.docx]

**S1 Table. Raw data for the maxillary sinus volumetry by automatic and manual quantifications.**

|  | **Total maxilary sinus volume (cm³)** | | | | **Air-free maxilary sinus volume (cm³)** | | | |
| --- | --- | --- | --- | --- | --- | --- | --- | --- |
|  | **Automatic tool** | | **Manual segmentation** | | **Automatic tool** | | **Manual segmentation** | |
| **Patient** | **Right MS** | **Left MS** | **Right MS** | **Left MS** | **Right MS** | **Left MS** | **Right MS** | **Left MS** |
| 1 | 20,15 | 21,50 | 19,56 | 20,59 | 20,15 | 21,50 | 19,56 | 20,59 |
| 2 | 16,63 | 13,64 | 16,31 | 13,01 | 16,63 | 13,64 | 16,31 | 13,01 |
| 3 | 10,11 | 9,22 | 10,93 | 11,82 | 9,66 | 5,20 | 10,88 | 4,76 |
| 4 | 9,87 | 10,45 | 9,42 | 10,29 | 9,87 | 10,45 | 9,42 | 10,29 |
| 5 | 15,61 | 14,52 | 15,48 | 16,48 | 15,61 | 14,52 | 15,48 | 16,48 |
| 6 | 16,46 | 14,21 | 17,99 | 15,39 | 15,86 | 8,28 | 17,65 | 8,14 |
| 7 | 6,56 | 7,70 | 6,37 | 7,26 | 6,56 | 7,70 | 6,37 | 7,26 |
| 8 | 16,77 | 17,55 | 17,66 | 17,42 | 16,77 | 17,55 | 17,66 | 17,42 |
| 9 | 17,31 | 22,82 | 19,80 | 24,94 | 16,28 | 21,24 | 18,22 | 23,03 |
| 10 | 20,78 | 18,95 | 23,47 | 19,58 | 20,78 | 18,95 | 23,47 | 19,58 |
| 11 | 11,81 | 10,77 | 12,55 | 12,95 | 11,81 | 10,77 | 12,55 | 12,95 |
| 12 | 14,34 | 17,47 | 16,33 | 17,86 | 13,17 | 15,43 | 14,77 | 16,30 |
| 13 | 11,48 | 12,54 | 12,83 | 13,53 | 9,71 | 11,02 | 9,87 | 10,58 |
| 14 | 12,92 | 11,84 | 13,24 | 12,97 | 0,24 | 10,12 | 0,27 | 10,92 |
| 15 | 10,39 | 11,09 | 11,50 | 11,90 | 10,00 | 10,54 | 11,27 | 11,68 |
| 16 | 17,00 | 14,02 | 18,05 | 15,22 | 17,00 | 14,02 | 18,05 | 15,22 |
| 17 | 23,41 | 21,84 | 23,21 | 22,93 | 21,32 | 20,83 | 22,03 | 22,32 |
| 18 | 13,38 | 14,79 | 13,42 | 13,36 | 13,38 | 14,79 | 13,42 | 13,36 |
| 19 | 14,17 | 15,15 | 15,02 | 15,35 | 13,71 | 14,03 | 14,92 | 14,83 |
| 20 | 10,88 | 10,66 | 11,44 | 11,71 | 10,88 | 10,66 | 11,44 | 11,71 |
| 21 | 20,41 | 18,37 | 20,53 | 18,61 | 19,73 | 17,84 | 20,46 | 18,58 |
| 22 | 20,46 | 19,93 | 21,24 | 20,22 | 19,55 | 19,28 | 20,59 | 20,13 |
| 23 | 11,38 | 10,50 | 11,97 | 11,90 | 10,43 | 9,95 | 11,31 | 11,09 |
| 24 | 16,47 | 15,02 | 19,30 | 17,62 | 15,35 | 12,17 | 17,46 | 13,61 |
| 25 | 5,81 | 6,62 | 7,15 | 7,30 | 5,30 | 5,69 | 6,15 | 6,19 |
| 26 | 16,26 | 18,10 | 17,18 | 19,03 | 16,26 | 18,10 | 17,18 | 19,03 |
| 27 | 11,71 | 13,20 | 12,84 | 14,27 | 11,71 | 13,20 | 12,84 | 14,27 |
| 28 | 20,67 | 21,36 | 20,53 | 21,95 | 20,67 | 21,36 | 20,53 | 21,95 |
| 29 | 17,46 | 19,25 | 18,40 | 19,79 | 17,46 | 19,25 | 18,40 | 19,79 |
| 30 | 9,84 | 11,03 | 9,64 | 11,92 | 9,26 | 5,25 | 9,59 | 5,71 |
